# P53-derived hybrid peptides induce apoptosis of synovial fibroblasts in the rheumatoid joint

**DOI:** 10.18632/oncotarget.23268

**Published:** 2017-12-15

**Authors:** Shih-Yao Chen, Ai-Li Shiau, Chao-Liang Wu, Chrong-Reen Wang

**Affiliations:** ^1^ Section of Rheumatology, Department of Internal Medicine, National Cheng Kung University Hospital, Tainan, Taiwan; ^2^ Department of Microbiology and Immunology, National Cheng Kung University Medical College, Tainan, Taiwan; ^3^ Department of Biochemistry and Molecular Biology, National Cheng Kung University Medical College, Tainan, Taiwan

**Keywords:** apoptosis, p53-derived hybrid peptide, p73, rheumatoid arthritis, synovial fibroblast

## Abstract

Loss of p53-mediated suppression by its dominant-negative counterpart is commonly observed in human cancers, and activating p73 is a therapeutic strategy in p53-mutated oncological patients. In synovial fibroblasts (SFs) from rheumatoid arthritis (RA), mutant p53 can lead to the transformation-like features with resistance to the apoptosis induction. We examined whether intra-articular (i.a.) administration of p53-derived hybrid peptides to activate p73 can induce apoptosis of SFs by using adenoviral vectors encoding 37 amino acid (Ad37AA), a p53-derived hybrid peptide capable of activating p73, to transduce SFs *in vitro* and inject collagen-induced arthritis (CIA) joints *in vivo*. Increased p73 expression was found in synovial lining layers and SFs of RA patients and CIA rats. Higher expression of p53 up-regulated modulator of apoptosis (PUMA) and Bax with enhanced apoptosis were found in Ad37AA-transduced SFs, and silencing p73 abrogated the up-regulation of PUMA and Bax. Articular indexes and histologic scores were reduced in Ad37AA-injected joints with decreased SF densities, increased apoptotic cell numbers, and higher PUMA expression levels. We demonstrate that i.a. administration of p53-derived hybrid peptides can activate p73 to induce apoptosis of SFs and ameliorate the rheumatoid joint, implicating an enhancement of the p73-dependent apoptotic mechanism as a pharmacological strategy in the RA therapy.

## INTRODUCTION

Although wild-type p53 can prevent the onset and development of tumor cells through activating the suppressive responses, its function loss due to a dominant-negative regulation from the mutant counterpart is a common feature in human cancers [1, 2]. Activating p73, another p53 family member, is a therapeutic strategy in p53-mutated oncological patients due to its rare mutation and redundant functions with wild-type p53 [3, 4]. In rheumatoid arthritis (RA), apoptosis-resistant synovial fibroblasts (SFs) constitute a major cell population of pannus tissues in the hyperplastic synovial lining layers, causing cartilage erosion and joint destruction [5, 6]. In particular, similar to the role in tumor *in situ*, p53 mutation in RASFs can lead to the transformation-like features with resistance to apoptosis induction [7]. Our previous experiments demonstrate that wild-type p53 is inactivated by its mutant counterpart in SFs from RA patients and collagen-induced arthritis (CIA) rats [8]. Nevertheless, expression of p73, related pathogenic mechanism and therapeutic implication remain unexplored in the rheumatoid joint. Inhibitory apoptosis stimulating protein of p53 (iASPP) can regulate the p53 family member-induced apoptosis by controlling the transcriptional activity of apoptosis-related molecules [9]. Interestingly, in p53-mutated or missing tumor cells, iASPP can bind p73 and inhibit apoptosis, and 37 amino acid (37AA), a p53-derived hybrid peptide derived from conserved box II (residues 118-142) and III (residues 171-181), dissociates such a binding and reverses the inhibition [10, 11]. Notably, intra-articular (i.a.) gene transfer can deliver intracellular acting molecules with sustained therapeutic efficacy and fewer extra-articular adverse effects [12, 13]. In this study, we examined whether transducing SFs and injecting CIA joints with the adenoviral vector encoding 37AA (Ad37AA) can dissociate the binding with iASPP to activate p73 and induce apoptosis. Our results demonstrated increased p73 levels with co-localized iASPP expression in synovial lining layers and SFs from the rheumatoid joint. Higher expression of p53 up-regulated modulator of apoptosis (PUMA) and Bax with enhanced apoptosis were found in Ad37AA-transduced SFs with lower iASPP-associated p73 levels, and silencing p73 abrogated the up-regulation of PUMA and Bax. Articular indexes and histologic scores were reduced in Ad37AA-injected CIA joints with decreased SF densities, increased apoptotic cell numbers, and higher PUMA expression levels.

## RESULTS

### Increased p73 levels with co-localized iASPP expression in the rheumatoid joint

**Figure 1:**
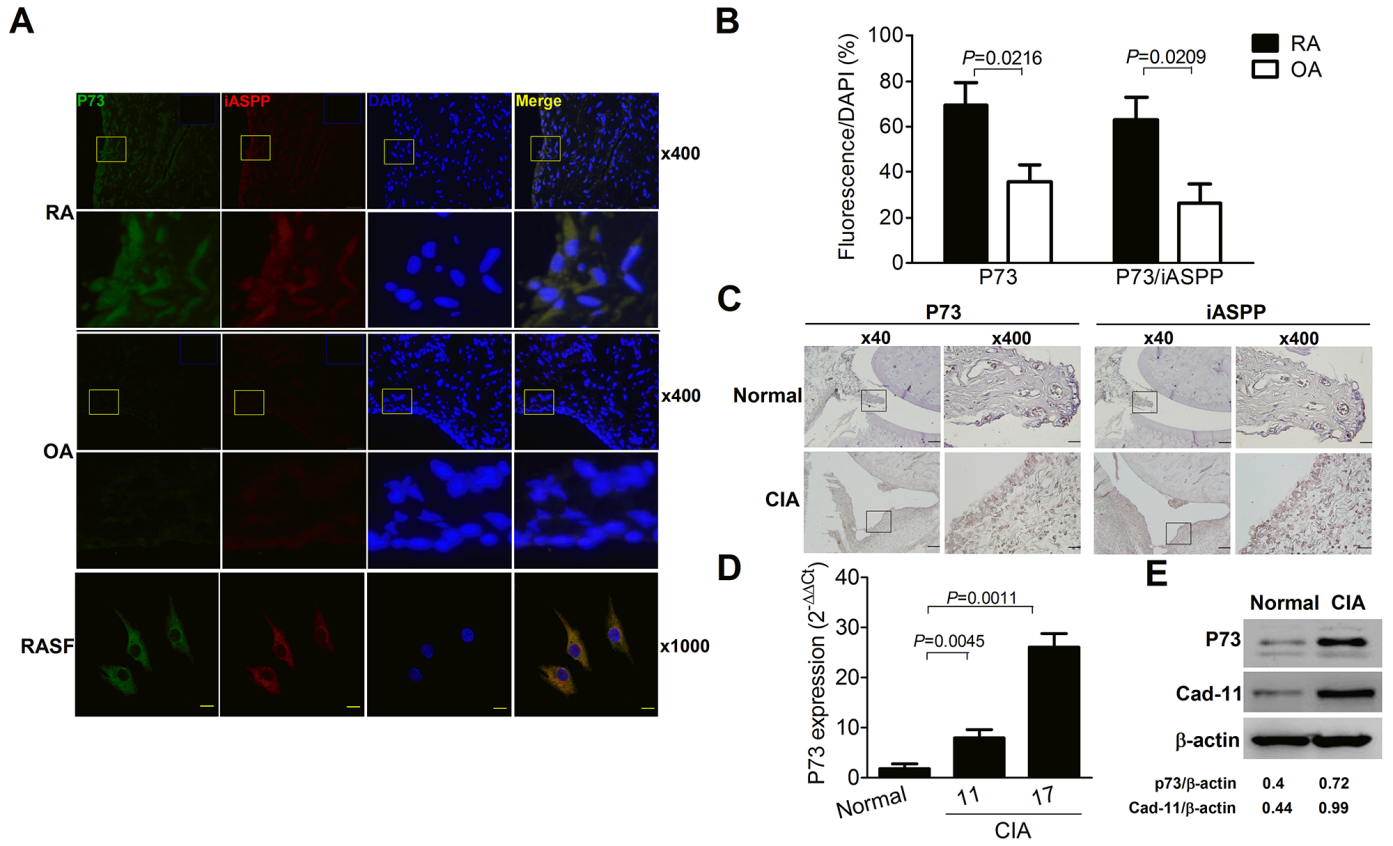
Increased p73 levels with co-localized iASPP expression in the rheumatoid joint **(A)** Immunofluorescence staining of p73 (green, FITC), iASPP (red, Texas red) and nucleus (blue, DAPI) in RA and OA synovial tissues (×400 magnification). The yellow-boxed areas in upper panels are magnified and shown in lower panels (×1,600 magnification). The blue-rectangled areas in the right upper corner of upper panels are isotype control IgG staining of p73 and iASPP. Immunofluorescence staining of p73 (green, Alexa Fluor 488), iASPP (red, Alexa Fluor 594) and nucleus (blue, DAPI) in RASF (×1,000 magnification, scale bar = 20 μm). **(B)** p73- or p73/iASPP double positive frequencies of DAPI-positive cells in synovial tissues. Each value represents the mean and SEM (n = 7). **(C)** Immunohistochemical detection of p73 and iASPP in synovial tissues of normal and CIA rats. Scale bars represent 500 and 50 μm in ×40 and ×400 magnifications, respectively. The boxed areas in left panels are magnified and shown in right panels. **(D)** Quantitative real-time PCR analysis of p73 levels from synovial tissues of normal and CIA rats. Each value represents the mean and SEM (n = 5). **(E)** Representative immunoblot photographs of p73 and cadherin-11 (Cad-11) expression in SFs from normal and CIA rats. The results in A to E are representative of at least two independent experiments with similar findings.

At first, synovial expression levels of p73 and iASPP in arthritis patients were examined by the immunofluorescent staining. Figure 1A demonstrates the intracellular expression of p73 and iASPP in the synovial lining layers of RA patients and osteoarthritis (OA) counterparts, and a representative staining pattern in RASFs with the distinct co-localization of both molecules. Further quantitation reveals increased frequencies of p73- and p73/iASPP double positive synoviocytes in RA patients as compared with OA counterparts (Figure 1B). Next, we examined the experimental CIA model. Representative immunohistochemical photographs in Figure 1C show higher expression of p73 and iASPP in the synovial lining layers. In Figure 1D, p73 levels were increased from day 11 onward and there were maximal expression levels of p73 on day 17 by the quantitative PCR analysis of synovial tissues. Figure 1E demonstrates higher expression levels of cadherin-11, a specific SF marker [14], and p73 in the lysates of SFs from CIA joints.

### Induction of apoptosis in SFs by transducing Ad37AA to activate p73

**Figure 2:**
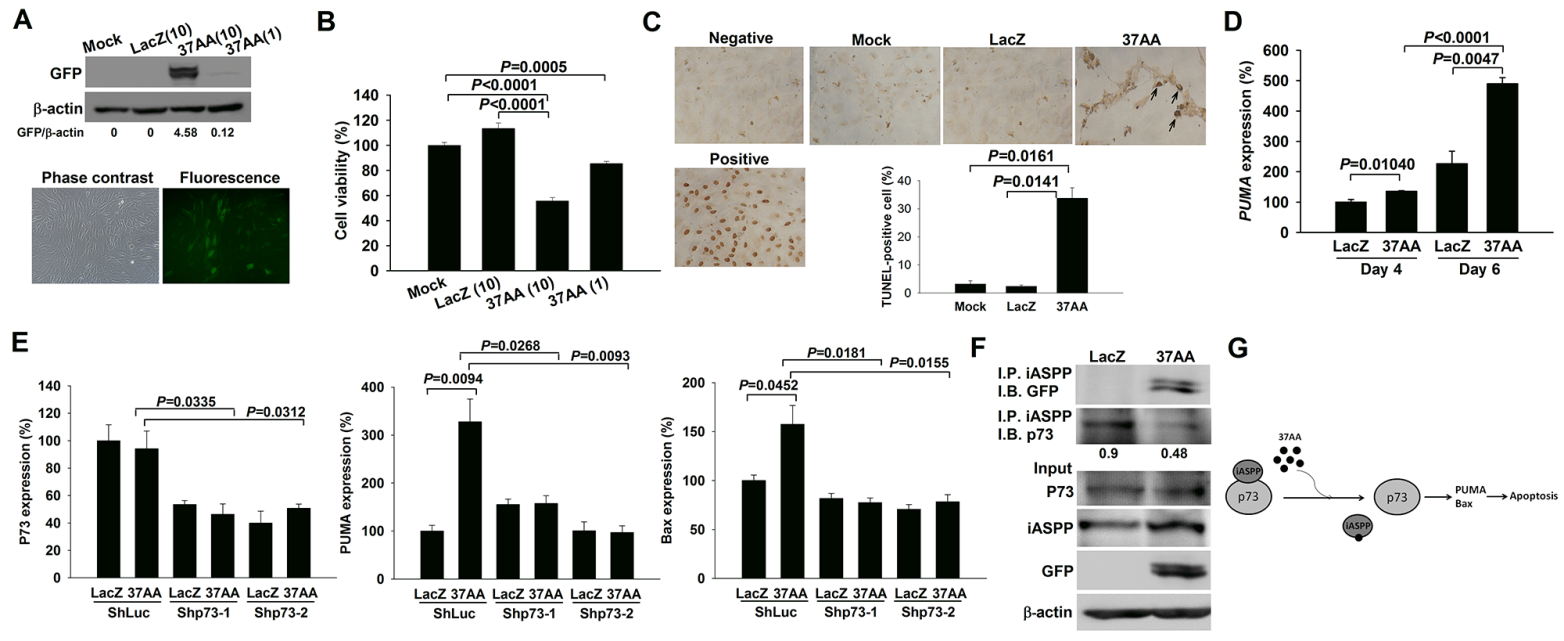
Induction of apoptosis in SFs by the activation of p73 **(A)** Immunoblot and immunofluorescence analyses reveal a high efficacy of Ad37AA transfection in CIASFs with expression of GFP protein and green fluorescence, respectively. **(B)** Cell viability is expressed as the percentages of surviving cells relative to those in mock cells. Each value shown in B represents the mean and SEM (n = 6). **(C)** TUNEL-positive cells were observed after the transfection of Ad37AA at a MOI of 10 for 7 days. DNase-treated cells were used as a positive control. Arrows indicate positive cells. Percentages were determined by averaging TUNEL-positive cells in 3 randomly selected fields at the ×100 magnification (n = 3). **(D)** Quantitative real-time PCR analysis demonstrates PUMA expression levels in Ad37AA-transduced CIASFs for 4 and 6 days. Each value shown represents the mean and SEM (n = 8). **(E)** Quantitative real-time PCR analysis reveals p73, PUMA and Bax expression levels in p73-silenced and control CIASF transfectants transduced with Ad37AA or AdLacZ for 6 days (n = 5). **(F)** Representative immunoblot photographs of iASPP-associated p73 expression in Ad37AA-treated CIASFs. Input represents the loading control. AdLacZ-transfected CIASFs were served as a control group in A to D. **(G)** A schematic diagram shows the dissociation of the binding with iASPP by overexpressing 37AA to activate p73 and induce apoptosis in SFs. The results in A to F are representative of at least two independent experiments with similar findings.

CIASFs were transduced with Ad37AA for 7 days, and the 37AA expression was verified by the immunoblot analysis and fluorescence microscopic observation (Figure 2A). Cell viability was reduced in Ad37AA-transduced CIASFs (Figure 2B) with increased apoptosis (Figure 2C) in comparison with the control counterparts. The expression of PUMA, a downstream molecule of the p73 signaling pathway [15], was examined to evaluate whether the enhanced apoptotic status is due to the activation of p73. Transducing CIASFs with Ad37AA increased the PUMA expression levels as compared with the control vectors (Figure 2D). There was an up-regulated expression of BAX, a Bcl-2 family member [16], after the Ad37AA transduction. Similar to the expression of PUMA, silencing p73 abrogated such an up-regulation, suggesting that other pro-apoptotic molecules like BAX are also involved in the p73-dependent 37AA-induced apoptosis (Figure 2E). Indeed, this finding is different from the previous observation by using cancer cells with the p53-null status to demonstrate that Ad37AA transduction results in significant activation of PUMA but has no effects on Bax [10]. To further verify that p73 activation was caused by dissociating the binding with iASPP, Ad37AA-transduced CIASFs were immunoprecipitated with anti-iASPP, followed by immunoblotted with anti-p73. There were significantly decreased iASPP-associated p73 levels, whereas total levels of p73 and iASPP were not altered in the input controls (Figure 2F). Collectively, a schematic diagram in Figure 2G demonstrates that the adenoviral vector-mediated overexpression of 37AA can dissociate p73 from the binding with iASPP to activate the downstream PUMA/Bax and induce apoptosis in p53-mutated SFs.

### Amelioration of CIA by the i.a. injection of Ad37AA to activate p73

**Figure 3:**
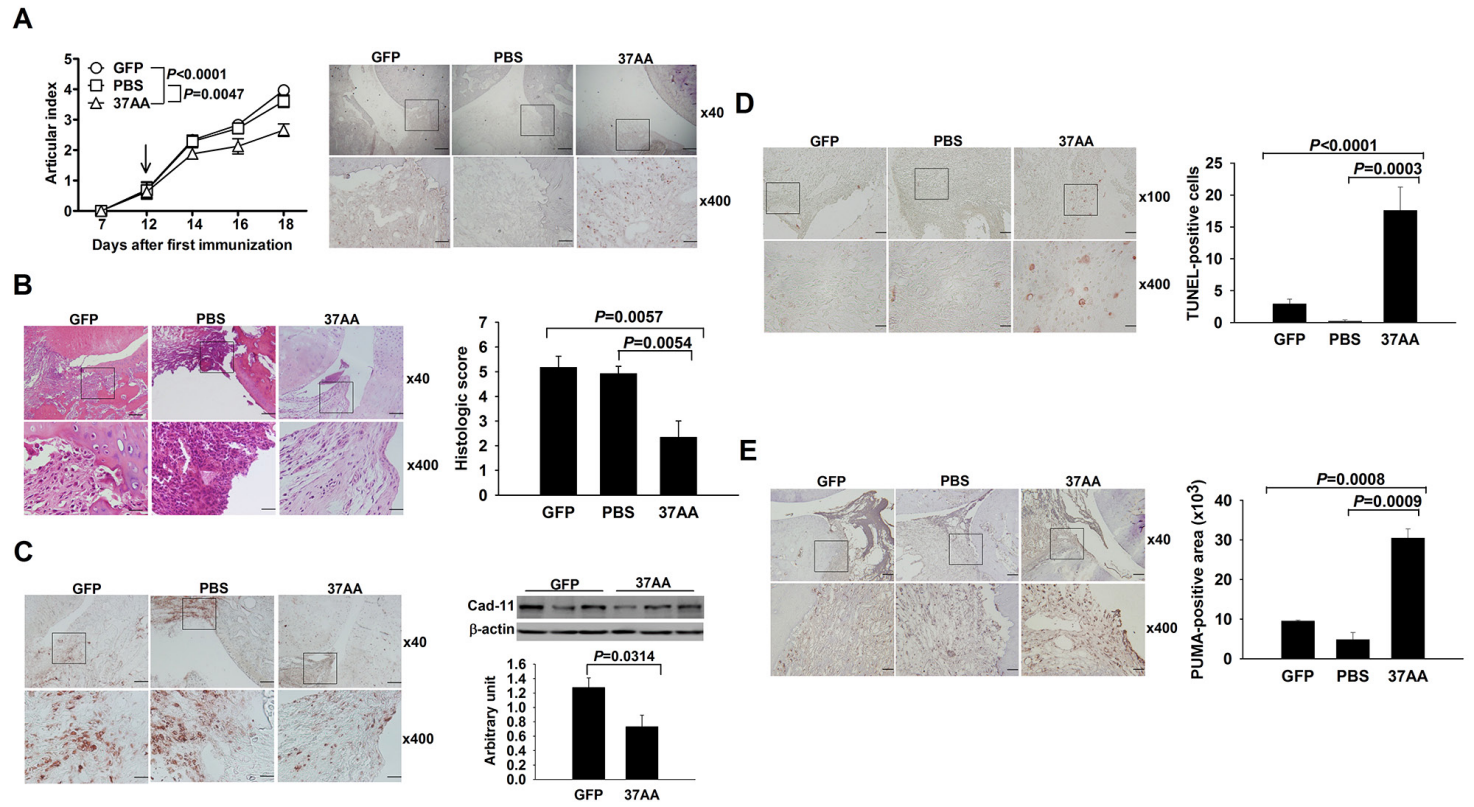
Amelioration of experimental arthritis by i.a. activation of p73 **(A)** Left, Reduced articular indexes in Ad37AA-injected CIA joints as compared with AdGFP (*P* < 0.0001) or PBS (*P* = 0.0047) control joints. Right, Representative GFP immunohistochemical photographs of CIA joints. **(B)** Representative H&E photographs and histologic scores in Ad37AA-injected CIA and control joints. **(C)** Left, Representative cadherin-11 (Cad-11) immunohistochemical photographs of CIA joints. Right, Immunoblot analysis of Cad-11 in CIA synovial extracts with quantification of the band intensity by comparing with β-actin. **(D)** Representative *in situ* apoptosis photographs and calculated TUNEL-positive cell numbers in CIA joints. **(E)** Immunohistochemical intensity with representative photographs of PUMA expression in CIA joints. CIA joints receiving different treatments were obtained on day 18 upon sacrifice. Scale bars represent 500, 200, and 50 μm in ×40, ×100 and ×400 magnifications, respectively. Each value in the graphs represents are mean and SEM (n = 8 in A and B, n = 3 in C, n = 5 in D and E). The results in A to E are representative of two independent experiments with similar findings.

Under the hypothesis that activation of p73 can induce apoptosis in SFs, we injected CIA joints with Ad37AA vectors. Expression of adenoviral vectors was demonstrated by the GFP staining in synovial tissues, and the Ad37AA-injected joints had smaller articular indexes in comparison with the control joints (Figure 3A). There were lower histological scores with less synovial hyperplasia and fewer erosions on the cartilage and bone (Figure 3B). Notably, Ad37AA-injected joints displayed decreased cadherin-11 expression levels by immunohistochemical and immunoblot analyses (Figure 3C). Furthermore, the TUNEL assay was used to examine whether the apoptotic process is responsible for reduced SF densities in CIA joints. Increased apoptotic cell numbers were identified in Ad37AA-injected joints as compared with the control ones (Figure 3D). In addition, the synovial expression of PUMA was analyzed to verify that induction of apoptosis is through the activation of p73. There were higher PUMA expression levels in Ad37AA-injected CIA joints (Figure 3E).

## DISCUSSION

The ability of RASFs to survive in the environment rich in apoptosis-inducing factors resembles the phenotype of selected tumor cells [6]. Previous data have shown that these cells express higher levels of mutant p53 capable of exerting a dominant-negative regulation on the wild-type counterpart, resulting in the loss of apoptosis-induced function [6, 8]. In this study, we identified abundant p73-positive synoviocytes localized at the synovial lining layers of RA and CIA joints. Although the functional redundancy exists between p73 and wild-type p53, in p53-mutated or missing cancer cells, the binding of iASPP with p73 prevents its activation to exert the functional role [3, 10]. By analyzing the RA synovial tissues and purified SFs, an intracellular co-localizaion of p73 and iASPP was clearly demonstrated in such cells. Further experiments were performed to dissociate the binding of p73 with iASPP by using the Ad37AA for *in vitro* transduction in CIASFs and *in vivo* injection into CIA joints. Both approaches activated p73 and raised the downstream expression of PUMA, resulting in the enhancement of apoptosis in SFs. Indeed, these findings provide evidences that dissociating the binding with iASPP molecule to activate p73 can induce the apoptosis of p53-mutated SFs.

Expansion of SFs in synovial lining layers is derived from inhibiting pro-apoptotic processes with distinct pathways responsible for their induction [16, 17]. Although RASFs express the extrinsic pathway-related death receptors, these cells are relatively resistant to apoptosis mediated by the exogenous stimulation through such receptors, TNF in particular. Furthermore, there is an increasing understanding of regulatory mechanisms responsible for the downstream signals to prevent apoptosis, and accumulated evidences indicate that the activated PI3K/Akt pathway plays an inhibitory role in the TNF-mediated apoptosis. Indeed, our previous experiments demonstrate that *in vitro* transducing SFs and *in vivo* injecting CIA joints with adenoviral vector encoding PTEN molecule can enhance apoptosis through the reduction of Akt activation [18]. The intrinsic pathway, largely independent of surface receptors, can be triggered by DNA damages in the rheumatoid joint with upregulated expression of wild-type p53 [6, 8]. Regarding the resistance to apoptotic stimuli in RASFs, the existence of mutant counterpart to block the downstream signaling machinery of wild-type p53 can be overcome by the forced expression of PUMA [19]. In this study, dissociating the iASPP binding to activate p73 could enhance the apoptosis of SFs through an upregulation of PUMA expression. Indeed, targeting SFs in the rheumatoid joint is a therapeutic strategy without interfering with the host defense against infection raised by the prevailing usage of biologics [5, 6]. A better insight into the molecular mechanisms related to apoptosis induction in SFs would allow the development of novel therapeutics in RA patients.

In conclusion, we demonstrate that i.a. administration of p53-derived hybrid peptides can activate p73 to induce apoptosis of SFs and ameliorate the rheumatoid joint, implicating an enhancement of the p73-dependent apoptotic mechanism as a pharmacological strategy in the RA therapy.

## MATERIALS AND METHODS

### Clinical samples from arthritis patients

Synovial tissues and purified SF were obtained from RA and OA patients with the informed consent from all subjects under the permission of Institutional Review Board of National Cheng Kung University Hospital (NCKUH).

### Construction of adenoviral vectors

The pShuttleCMV-37AA, made by inserting GFP and 37AA fragments into pShuttleCMV (Stratagene) by using BglII and NotI, was cut by *Pme*I into linearized vector and cotransfected with adenoviral backbone vector pAdeasy-1 (Stratagene) into BJ5183 strain. The generated recombinant plasmid Ad37AA was digested with *Pac*I and transfected into 293 cells to obtain the high-titer adenoviral vectors, as previously described [8]. Adenoviral vectors encoding β-galactosidase (AdLacZ) and green fluorescence protein (AdGFP) were used as the control vectors in following experiments.

### Lentiviral vectors and p73-silenced SF transfectants

The p73 shRNA-expressing pLKO.1-shp73-1 (TRCN0000272587) and -2 (TRCN0000284787) as well as luciference shRNA-expressing pLKO.1-shLuc (TRCN0000072246) lentiviral plasmids were obtained from the National RNAi Core Facility (Academica Sinica, Taiwan). Recombinant lentiviral vectors, LVshp73-1, LVshp73-2 and LVshLuc, were produced by transiently transducing 293T cells with pLKO.1-shp73-1, pLKO.1-shp73-2 and pLKO.1-shLuc, respectively, together with packing plasmids psPAX2 and envelope plasmids pMD2G by the calcium phosphate precipitation method, as previously described [21]. To produce stable transfectants where p73 is silenced and control ones, CIASFs were transduced with LVshp73-1, LVshp73-2 and LVshLuc for 48 hrs, respectively, under 8 μg/mL polybrene (Sigma-Aldrich), followed by selection with incubation of puromycin (2 μg/mL) for 2 wks, as previously described [20].

### Cell viability and TUNEL assay

SFs were prepared from CIA joints upon killing on day 18, as previously described [8, 20]. CIASFs (5×10^3^/well) in 96-well dishes were transduced with AdLacZ or Ad37AA at a total multiplicity of infection (MOI) of 1 or 10, or left untreated for 7 days. Apoptotic cells were evaluated by the TUNEL assay (Promega), and cell viability was determined by the colorimetric WST-1 assay (TaKaRa).

### Quantitative real-time polymerase chain reaction (PCR)

After the total RNA isolation with synthesis of cDNA from synovial tissues, SFs or SF transfectants, the real-time PCR was performed to quantify p73, PUMA, Bax and GAPDH levels by using SYBR Premix Ex Taq (TaKaRa) in the SmartCycler (Cepheid). Primer sequences were forward: 5′-TCCCTTCCAACACCGACTACCCT -3′/reverse: 5′-GAGGTGGTGGTGTGGACACTTTGA-3′, forward: 5′-CAGCAGCACCTAGAGTCGCCCG-3′/reverse: 5′-CTCTTCTTGTCTCCGCCGCTCGTA-3′, forward: 5′-ATGGAGCTGCAGAGGATGATTGC-3′/reverse: 5′-GCACAGGGCCTTGAGCACCA-3′, and forward: 5′-CTCATGACCACAGTCCATGCCATC-3′/reverse: 5′-CGTTCAGCTCTGGGATGACCTTG-3′, respectively. The PCR profile contained an activation step at 95°C for 2 min, followed by 40 cycles with 10 sec at 95°C, 10 sec at 59°C and 15 sec at 72°C. The comparative Ct method was used to calculate the relative abundance of p73 and PUMA as compared with GAPDH expression.

### CIA induction and i.a. transfer of Ad37AA

Male 8-week old Sprague-Dawley rats were immunized with bovine type II collagen emulsified with complete Freund’s adjuvant on days 0 and 7, as previously described [8, 20]. Animal experiments were approved by the Institutional Animal Care and Use Committee of NCKU. Each joint received the i.a. injection of 5 × 10^7^ plaque-forming units (PFU) of Ad37AA and AdGFP into right and left ankles on day 12, respectively with PBS injection as another control group. Arthritis was graded clinically with articular indexes, and hematoxylin and eosin (H&E)-stained joint sections were evaluated by histological scores, as previously described [8, 20].

### Immunohistochemical, *in situ* apoptosis and immunofluorescent analyses

Processed paraffin-embedded synovial sections were stained with anti-GFP (Santa Cruz), anti-PUMA (Santa Cruz), anti-p73 (Santa Cruz), anti-iASPP (Santa Cruz), anti-cadherin-11 (Cell Signaling) or isotype control IgG (Santa Cruz), followed by secondary antibodies and substrate chromogen with expression intensities quantitated by the HistoQuest software (Tissue Gnostics). Apoptotic cells in cryostat synovial sections were detected by the TUNEL assay (Promega). For immunofluorescent analyses, pre-treated paraffin-embedded synovial sections were incubated with anti-p73 (Santa Cruz), anti-iASPP (Santa Cruz), or isotype control IgG (Santa Cruz), followed by FITC- and Texas red-conjugated secondary antibodies (BD Biosciences), respectively, and observed by the fluorescence microscopy. Apoptotic and fluorescence-positive cells were counted by averaging their numbers in 3 randomly selected fields at the ×400 magnification, as previously described [18]. Purified SFs were subjected to anti-p73 (Santa Cruz) and anti-iASPP (Santa Cruz), followed by Alexa Fluor 488- and Alexa Fluor 594-conjugated secondary antibodies (Life Technologies), respectively, and observed by the confocal microscopy.

### Immunoblot and immunoprecipitation analyses

Lysates of SFs or extracts from synovial tissues were subjected to immunoblot with anti-p73 (Santa Cruz), anti-GFP (Santa Cruz), or anti-cadherin-11 (Cell Signaling), and the blots were re-probed with anti-β-actin (Sigma-Aldrich) as a quantitative control, as previously described [8, 20]. AdLacZ or Ad37AA-transduced SF lysates were immunoprecitated with anti-iASPP (Santa Cruz), followed by immunoblotted with anti-p73 (Santa Cruz) or anti-GFP (Santa Cruz), and small quantities (10%) were snapped for immunoblot analysis as an input control.

### Statistical analysis

Data are expressed as the mean ± SEM. Differences in frequencies of p73- and p73/iASPP double positive synoviocytes between RA and OA patients were compared by Mann-Whitney U test. Statistical significance in articular indexes was compared by the repeated-measures analysis of variance. The remaining data was assessed with Student’s *t*-test. *P* values less than 0.05 were considered significant.
